# Evaluation of T-Cell Responses Following Sequential Vaccination with PCV13 and PPSV23 Against *Streptococcus pneumoniae* in Patients with Psoriasis

**DOI:** 10.3390/vaccines13090920

**Published:** 2025-08-29

**Authors:** Thea Wojtakowski, Lukas van de Sand, Lorena Helmer, Mona Mokanis, Oliver Witzke, Peter A. Horn, Adalbert Krawczyk, Wiebke Sondermann, Monika Lindemann

**Affiliations:** 1 Institute for Transfusion Medicine, University Hospital Essen, University Duisburg-Essen, 45147 Essen, Germany; t.l.wojtakowski@gmail.com (T.W.); peter.horn@uk-essen.de (P.A.H.); 2 Department of Infectious Diseases, University Hospital Essen, University Duisburg-Essen, 45147 Essen, Germany; lukas.vandesand@uk-essen.de (L.v.d.S.); lorenahelmer@hotmail.de (L.H.); mona.mokanis@uk-essen.de (M.M.); oliver.witzke@uk-essen.de (O.W.); adalbert.krawczyk@uk-essen.de (A.K.); 3 Institute for Virology, University Hospital Essen, University Duisburg-Essen, 45147 Essen, Germany; 4 Department of Dermatology, Venereology and Allergology, University Hospital Essen, University Duisburg-Essen, 45147 Essen, Germany; wiebke.sondermann@uk-essen.de

**Keywords:** *Streptococcus pneumoniae*, psoriasis, cellular immunity, ELISpot, IFN-γ, sequential vaccination

## Abstract

Background: Characterization of cellular responses to vaccinations in immunocompromised patients remains an evolving area of research. This particularly applies for pneumococcal vaccination in diseases such as psoriasis and in the setting of immunosuppressive therapy. Methods: This prospective study included 42 patients with moderate-to-severe psoriasis. Following German guidelines at the time, patients underwent a sequential vaccination protocol against *Streptococcus pneumoniae*, consisting of Prevenar 13 (PCV13) and Pneumovax 23 (PPSV23). Over a 7-month period, we analyzed T-cell responses to common serotypes of *Streptococcus pneumoniae* using an interferon-γ ELISpot assay. For comparison, we performed an ELISA to measure pneumococcus-specific antibody production. Results: Patients undergoing anti-TNF-α blocker therapy, monoclonal antibody therapy (specifically anti-IL-12/23, IL-23, and IL-17), and methotrexate therapy showed significantly different responses to the pneumococcal serotype PS14 at onset (*p* = 0.02). T-cell responses ranged from strong (PS9N, PS14, PS25F) and intermediate (PS2) to weak (PS6A and PS11A). We did not observe a significant correlation of IgG antibodies with the magnitude of cellular immune responses. Conclusions: Immunosuppressive therapy alters vaccination-induced cellular immunity in psoriasis patients. Further research is needed to clarify the mechanisms involved.

## 1. Introduction

Psoriasis is one of the most common inflammatory dermatoses, affecting approximately 2% of the German population [1]. According to current understanding, psoriasis is a chronic, systemic, immune-mediated inflammatory disorder comprising three major domains: the skin, the bones/joints, and the blood vessels, all characterized by a similar pattern of inflammation [2].

Apart from these major domains, psoriasis is associated with a number of concomitant conditions, especially belonging to the spectrum of metabolic syndrome, referred to as comorbidity [3]. Psoriasis has a profound impact on the quality of life of those affected, largely due to its chronic nature and the visibility of skin lesions [4]. The severity of the disease is typically assessed using objective measures such as the Psoriasis Area and Severity Index (PASI) and/or Body Surface Area (BSA), as well as the Dermatology Life Quality Index (DLQI) as a patient-reported outcome [5]. Based on these measures, mild psoriasis is usually defined as a PASI of ≤10 and/or BSA of ≤10% and DLQI of ≤10, whereas moderate-to-severe psoriasis is usually defined as a PASI or BSA of >10 and DLQI of >10. Special clinical situations can change mild psoriasis to moderate-to-severe, including involvement of so-called high-impact areas, which is associated with a high subjective burden [6,7]. The pathophysiology of psoriasis involves the excessive release of pro-inflammatory mediators, including tumor necrosis factor-alpha (TNF-α) as well as interleukin (IL)-23 and -17 as a central mechanism.

Current guidelines recommend systemic therapy for moderate-to-severe cases, including immunosuppressive or immunomodulatory agents such as fumaric acid esters, methotrexate (MTX), the small molecule apremilast (a phosphodiesterase-4 inhibitor), and a range of biologic therapies. The first class of biologics introduced for psoriasis treatment was TNF-α inhibitors, including adalimumab, etanercept, infliximab, and certolizumab. More recent biologics selectively target interleukins—either IL-12/23 (ustekinumab), IL-23 (guselkumab, tildrakizumab, risankizumab), or IL-17 (secukinumab, ixekizumab, bimekizumab, and brodalumab) [8,9].

While systemic therapies are effective in managing psoriasis, their immunosuppressive effects—particularly their impact on cytokine expression in T-cells—combined with the high prevalence of comorbidities, increase the vulnerability of patients to infections, including pneumococcal disease [9,10]. These remain a significant global health burden, leading to substantial morbidity and mortality, particularly in vulnerable populations [9,10,11,12]. Streptococcus pneumoniae is, despite vaccination campaigns, the leading pathogen responsible for community-acquired pneumonia (CAP) [10].

Certain groups, including the elderly (>65 years), patients with significant comorbidities, and individuals with immune-mediated inflammatory diseases requiring immunosuppressive therapies, are at a higher risk for severe pneumococcal disease and its complications [13,14,15,16,17,18].

Vaccination is a cornerstone in the prevention of pneumococcal infection. Currently, a 20-valent conjugate vaccine (PCV20) is recommended for adults by the STIKO in Germany as of September 2023 [19]. At the time of data collection, two pneumococcal vaccines were routinely used in clinical practice: a 13-valent conjugate vaccine (PCV13)—based on the same principle as PCV20—and a 23-valent polysaccharide vaccine (PPSV23) [20].

PPSV23 induces a T-cell-independent immune response, resulting in the production of serotype-specific IgM and IgG2 antibodies, but it generates limited immune memory [15,21]. In contrast, PCV13 conjugates polysaccharides with a carrier protein, enabling a T-cell-dependent response and enhanced immunogenicity [14,21]. In Germany, national guidelines, published by the Robert-Koch Institute (RKI), recommend a sequential regimen of pneumococcal vaccination for individuals with chronic diseases and those undergoing immunosuppressive therapy. The former protocol included an initial dose of PCV13 followed by PPSV23 after 6 to 12 months [20].

Evaluating vaccination success in immunosuppressed patients is challenging due to the heterogeneity of immunosuppressive strategies tailored for different conditions [22,23,24]. For individuals with autoinflammatory diseases, data on pneumococcal antibody production are scarce, and studies investigating the cellular immune response to *S. pneumoniae* are even more limited. Most research on pneumococcal vaccine efficacy in immunocompromised patients, such as organ transplant recipients or patients post-splenectomy, has focused primarily on serological responses, with only a few exploring the cellular immune response after sequential vaccination [25,26].

Given the lack of specific data on psoriasis patients, understanding the cellular immunization process is critical, as it is central to developing immunological memory [27].

To accurately evaluate the vaccination success in patients with psoriasis receiving systemic treatments, it is essential to investigate T-cell-mediated immune responses. This approach is vital to provide a comprehensive understanding of immunization efficacy in this unique patient population and address the current knowledge gaps.

## 2. Materials and Methods

### 2.1. Study Population

**Figure 1:**
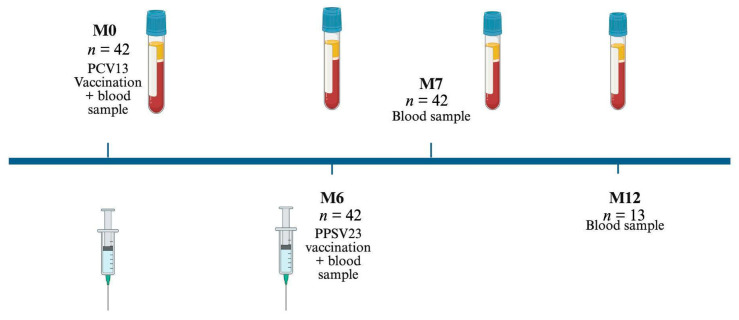
Study design. All patients sequentially receive a single dose of PCV13 and PPSV23. Blood samples are drawn at the indicated time points.

This single-center study enrolled *n* = 42 individuals diagnosed with moderate-to-severe psoriasis between March 2020 and December 2021, all of whom were undergoing active treatment (Table 1). Following official guidelines, patients received a single dose of Prevenar 13 prior to which the initial blood sample was collected for reference. Six months after the initial vaccination, an additional blood sample was collected, followed by administration of Pneumovax 23. Blood sample collection was subsequently repeated at the follow-up points 1 month and 6 months after the second vaccination (Figure 1). A follow-up sample 12 months after the second vaccination was analyzed in *n* = 13 patients.

Eligible participants were individuals aged 18 years or older, with no signs of active infections, and with clinically well-controlled disease activity. Exclusion criteria included pregnancy, change in therapy to another group, or pneumococcal vaccination within the last six years. Clinical endpoints such as pneumonia were monitored until month 27–36 (median 28) after the first vaccination.

Written informed consent was obtained from all participants. This study received ethical approval from the institutional review board of the University Hospital Essen (19-8670-BO) and was conducted in compliance with the Declarations of Helsinki and Istanbul and their subsequent amendments.

### 2.2. Vaccines

Two types of vaccines were used in the current study. Prenevar-13 contains polysaccharides from the serotypes 1, 3, 4, 5, 6A, 6B, 7F, 9V, 14, 18C, 19F, 19A, and 23F that are conjugated to a nontoxic diphtheria toxin cross-reactive material 197 protein, provoking a T-cell-mediated response. The vaccine contains 2.2 μg of each polysaccharide (except for 4.4 μg of serotype 6B), along with 5.0 mM succinate buffer, 0.85% sodium chloride, 0.02% polysorbate 80, and 0.125 mg of aluminum as aluminum phosphate per 0.5 mL dose [14].

Pneumovax-23 is a 23-valent polysaccharide vaccine containing the serotypes 1, 2, 3, 4, 5, 6B, 7F, 8, 9N, 9V, 10A, 11A, 12F, 14, 15B, 17F, 18C, 19F, 19A, 20, 22F, 23F, and 33F each at a dose of 25 μg in a formulation of phenol and <1 mmol sodium chloride per dose (0.5 mL) and is responsible for inducing a T-cell-independent response [15].

### 2.3. Measurement of Cellular Immunity Against S. pneumoniae

In order to assess the cell-mediated immunity after vaccination, an interferon-γ (IFN-γ) ELISpot assay was employed. Peripheral blood mononuclear cells (PBMCs) were collected from 9 mL of heparinized blood samples. The cells were isolated using density gradient centrifugation. After cell counting with an automated hematology analyzer (XP-300 Sysmex, Norderstedt, Germany), we pipetted 200,000 cells onto U bottom plates containing pneumococcal polysaccharides. These comprised the serotypes 2, 6A, 9N, 11A, 14, and 25F at three different concentrations (100, 150, and 200 μg/mL). Subsequently, a quantity of 150 μL AIMV medium (Gibco, Grand Island, NE, USA) was added to the triplicate cultures. For the three negative controls, cells were cultured without polysaccharides, and the T-cell mitogen phytohemagglutinin (PHA, 4 μg/mL) was used for positive controls. The duration of incubation was set at 24 h at a temperature of 37 °C and 5% CO_2_. Thereafter, cultures were transferred to precoated ELISpot plates (T-Track^®^ ELISpot kit, Mikrogen GmbH, Neuried, Germany) and once more incubated for 24 h. Following this, the plates were washed to remove cells and left to incubate for a period of two hours after application of a conjugate (5 μL) and dilution buffer (900 μL). Subsequently, 50 μL of substrate was added to the plates, which were then left in the dark for 6 min in order to allow spot formation. This process enabled the visualization of IFN-γ producing cells. For the purpose of quantification and automatic cell count, the ELISpot reader AID iSpot (AID Fluorospot, Autoimmun Diagnostika GmbH, Strassberg, Germany) was employed. The median values of responses to three polysaccharide concentrations were calculated, and the median responses of the negative controls from the ELISpot assay were considered when creating the spot increments. Three spots increments were set as a cutoff for positive responses. Negative controls remained under a mean spot count of 0.4, while positive controls were stable at a mean count between 233 and 308 at the three time points.

### 2.4. Pneumococcal Antibody ELISA

Peripheral venous blood samples were collected from participants under standardized conditions. After clotting at room temperature, the samples were centrifuged to isolate the serum. In order to evaluate the humoral immune response to pneumococcal vaccination, the VaccZyme Human Anti-PCP Enzyme Immunoassay (ELISA) (The Binding Site, Birmingham, UK) was employed. This commercial assay quantifies IgG antibodies against pneumococcal capsular polysaccharides in a serotype-independent manner. It measures the cumulative immune response to all 23 serotypes included in the 23-valent pneumococcal polysaccharide vaccine (PPSV23). To ensure specific detection of pneumococcus-targeted IgG, the serum was pre-absorbed with pneumococcal polysaccharide solutions, following the kit protocol, to remove non-specific antibody binding.

The ELISA method was performed in accordance with the manufacturer’s instructions. Serum samples, calibrators, and controls were added to microtiter wells pre-coated with pneumococcal polysaccharides. Following a 1 h incubation at 37 °C, unbound components were removed through multiple washing steps. Subsequently, a horseradish peroxidase (HRP)-conjugated anti-human IgG antibody was added, followed by a second incubation and additional washing. A tetramethylbenzidine (TMB) substrate was then applied, resulting in a colorimetric reaction proportional to the amount of bound antibodies. The reaction was halted with the addition of sulfuric acid, and the absorbances were measured at 450 nm using a microplate reader.

Pneumococcal serotype-specific IgG concentrations for six serotypes (2, 3, 6A, 9N, 11A, 14) were measured by serotype-specific ELISA as previously described by Mülling et al. using the WHO reference protocol [16].

### 2.5. Statistical Analysis

The data were examined with GraphPad Prism Version 10.5.0 (Graph Pad Software, Boston, MA, USA) or IBM SPSS Statistics version 25 (Armonk, NY, USA). The Shapiro–Wilk test was conducted to determine normal distribution of the data. Given that several variables showed non-normal distribution, non-parametric methods were employed to further analyze the data. ELISpot and ELISA data were assessed at different time points using the Kruskal–Wallis test, followed by Dunn’s multiple comparison test. The correlation between numerical variables was determined using the two-tailed Spearman test. In order to assess the effect of medication on IFN-γ production, the Kruskal–Wallis test was used. Results were considered significant at *p* < 0.05.

## 3. Results

### 3.1. Clinical Course of the Study Population

During the course of this study, no confirmed cases of hospitalized pneumonia were observed up until March 2023 (22 months after vaccination completion), with the exception of one case of confirmed lethal pneumonia, for which no microbiological evidence could be found and for which there was no evidence of a bacterial superinfection. The median age of the patients was 49 years (range 18–67 years). Initially, *n* = 61 patients were recruited. The data set for month 0 consisted of *n* = 61 measurements, the data set for month 6 consisted of *n* = 57 measurements, and the data set for month 7 consisted of *n* = 52 measurements. Of the total sample size of *n* = 61 patients, 42 complete data sets (month 0 to 7) were finally collected due to recruitment difficulties during the SARS-CoV-2 pandemic. Illness severity remained steadily low with a mean PASI of 2 at month 0 and month 6. Furthermore, mean leukocyte counts were found to be at an overall normal level, with a count of 7.39/nL at month 0 and 7.68/nL at month 6. There were no cases of leukopenia. The mean duration of therapy application at the time of vaccination was 26 months.

### 3.2. Cellular Immune Response to Pneumococcus-Specific ELISpot

We analyzed the cellular immune response at four different time points: prior to vaccination (month 0), 6 months after application of a single dose of Prevenar 13, 1 month after application of Pneumovax 23 (7 months after the initial vaccination), and 6 months after Pneumovax 23 (that is 12 months after starting the vaccination process). Of the serotypes used for in vitro tests in this study, three were included in Pneumovax 23 (PS2, PS9N, PS11A), PS6A was included in Prevenar 13, PS14 in both, and PS25F in none of the vaccines. Serotypes were chosen to demonstrate vaccine-specific responses and according to epidemiological importance. PS25F was added to show an immune response independent of vaccination.

To analyze the individual response to a specific serotype, the median and mean number of spots formed was calculated considering three pneumococcal polysaccharide concentrations (100 μg/mL, 150 μg/mL, and 200 μg/mL) (Table 2).

**Figure 2:**
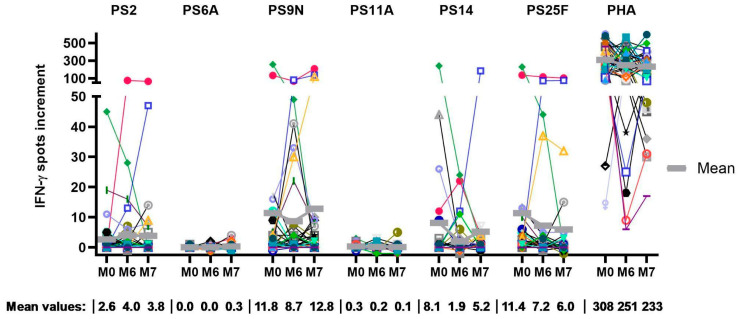
This graph depicts *S. pneumoniae-specific* T-cell responses following the vaccination protocol with PCV13 (M0), PPSV23 (M6), and the follow-up at month 7 (*n* = 42). Serotypes are chosen by inclusion or exclusion in either vaccine (PS6A in PCV13 and PS2, PS9N, PS11A in PPSV23), both (PS14), or none (PS25F). Cellular responses are given as spots increment; i.e., we choose median values of triplicate cultures and calculate the increment by subtracting negative controls from pneumococcus-stimulated values at each of the time points. The IFN-γ spot increments are displayed at a non-linear scale. Higher spot counts (>50) are compressed, for better visibility of lower values. Mean values are indicated by horizontal gray lines.

By calculating the IFN-γ spots increment, the mean spot formation over the time course was determined, and the highest response was observed for PS9N, PS14, and PS25F (Figure 2). A subgroup of patients even showed spots increment exceeding 100. Despite PS6A being unique to PCV13, an increase in cellular responses could not be observed, as the mean spot formation remained almost undetectable at all three time points. For the serotypes covered by PPSV23, PS9N induced a minor increase in cellular responses, with a mean spot increment of 8.6 before application of PPSV23 and a mean spot increment of 12.8 at month 7 (1.5-fold increase). Similarly, PS2 stimulation led to a minor change in mean spot increment (2.6 at baseline to 3.8 at month 7). The median values indicated that more than half of the patients showed no or a very minor response; i.e., the result was between 0 and 2.5 spot increment at all time points (Table 2 and Figure 2).

Similar to PS6A, a very low level of spot formation was observed after stimulation with PS11A, with mean values remaining below one spot increment throughout the observation period. In addition, almost no baseline activity could be recorded. Conversely, the mean results for PS14, a component of both vaccines, were between a 1.9 and 8.1 spot increments. After application of PPSV23, a 2.7-fold increase was observed; however, compared to baseline, the increase was only 1.1-fold. When looking at PS25F, which was not included in either of the vaccines, robust spot formation was recorded. The mean spot count ranged from 6.0 to 11.4 spots increment. For the positive controls, strong responses were achieved with spot increments over 200 in the vast majority of samples, during all four time points.

A small cohort of 13 individuals was tested 12 months after the start of the vaccination protocol. Due to the small sample size, only a limited analysis was performed. However, preliminary data indicate an increase in the mean and median values for most serotypes at month 12.

In summary, this study revealed a substantial cellular response to a subset of pneumococcal serotypes. Responses to serotype 9N, 14, and 25F were strong at all time points, responses to serotype 2 were intermediate, and responses to serotype 6A and 11A were weak. However, considering the total cohort of 42 psoriasis patients, no clear increase in cellular immunity after pneumococcal vaccination was identified.

### 3.3. Correlation of the Therapeutic Regimen with the Cellular Response

**Figure 3:**
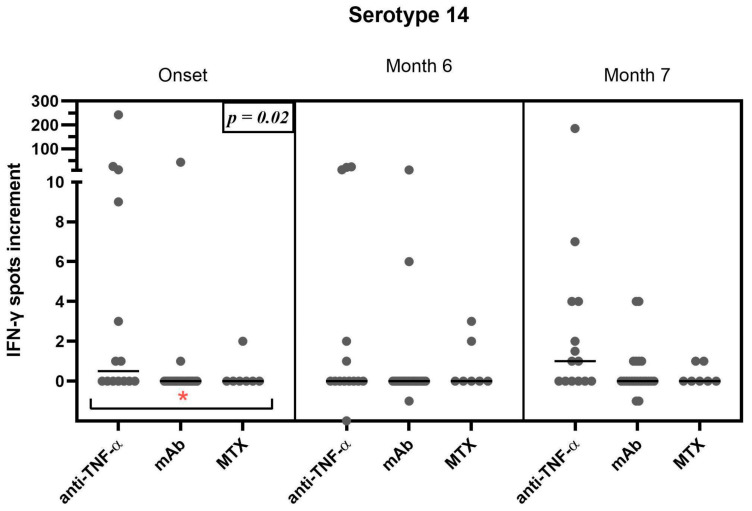
Therapy-specific cellular ELISpot response to serotype 14 over the course of surveillance. To assess each of the ELISpot responses, mean values of triplicate cultures (with antigen concentrations of 100, 150, and 200 μg/mL) are considered. Therapy regimens include TNF-alpha-blockers (*n* = 14), monoclonal antibodies (IL-12/23, IL-23, and IL-17-blockers) (*n* = 21), and methotrexate (*n* = 7). Mean values of each treatment group are shown as horizontal lines. Data are compared by the Kruskal–Wallis test, followed by Dunn’s multiple comparison test. Statistical significance is marked in the first column (Onset) for comparison of all three groups (* *p* = 0.02).

The impact of therapeutic regimens on ELISpot results was evaluated by the Kruskal–Wallis test, followed by Dunn’s multiple comparison test. Patients undergoing anti-TNF-alpha-blocker therapy (*n* = 14), monoclonal antibody therapy (specifically anti-IL-12/23, IL-23, and IL-17) (*n* = 21), and methotrexate therapy (*n* = 7) showed a significant difference at the onset of this study, when comparing the three groups (*p* = 0.02). However, responses to the serotype PS14 were not significantly higher in direct comparison for patients treated with TNF-alpha-blockers and MTX (*p* = >0.99) or TNF-alpha-blockers and monoclonal antibodies (*p* = 0.24) at onset. Subsequent comparisons at month 6 (*p* = 0.4) and month 7 (*p* = 0.7), however, did not demonstrate a significant impact of treatment on the vaccine response (Figure 3). Nevertheless, the TNF-alpha-treated cohort showed higher T-cell responses also at month 7. A rise in mean spot formation only occurred in patients under TNF-alpha-inhibitors from months 6 to 7 with a 3.5-fold increase. No significant differences were observed in the other cohorts and for the remaining serotypes

### 3.4. Correlation of the Therapeutic Regimen with the Humoral Response

**Figure 4:**
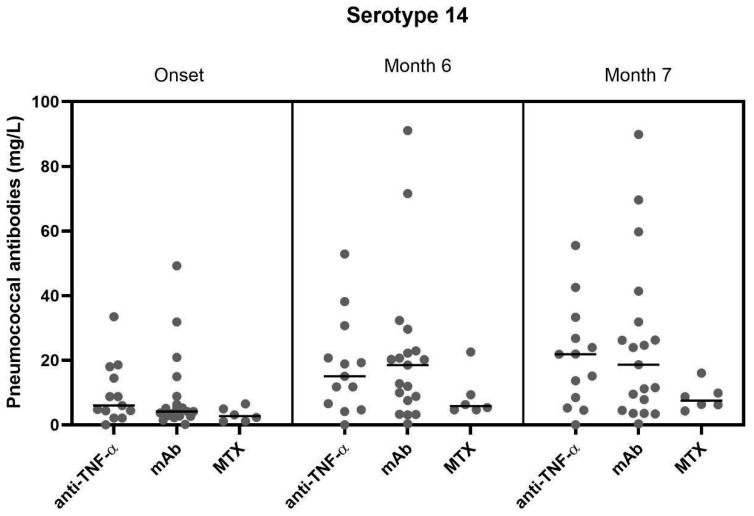
Therapy-specific pneumococcal antibody concentrations, directed against serotype 14. Antibody production is split by three different treatment groups (anti-TNF-alpha-blockers, monoclonal antibodies, and MTX). Mean values of each treatment group are shown as horizontal lines. Data are compared by the Kruskal–Wallis test, followed by Dunn’s multiple comparison test with *p* values showing non-significance.

Similar to the cellular data, the impact of therapeutic regimens on pneumococcal antibody results was evaluated by the Kruskal–Wallis test, followed by Dunn’s multiple comparison test (Figure 4). Unlike Figure 3, the groups treated with TNF alpha-blockers and monoclonal antibodies tended to show similar and relatively high mean antibody concentrations across all time points, while the group treated with MTX showed lower responses at all time points. The comparison of the individual groups at all three time points was assessed as non-significant.

### 3.5. Correlation of Cellular Immune Responses and Antibody Production

**Figure 5:**
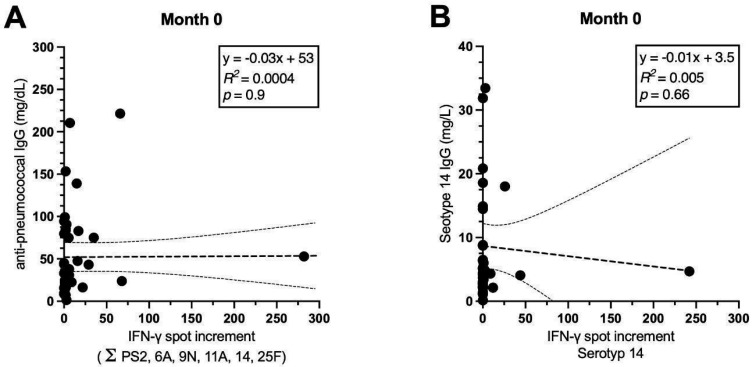

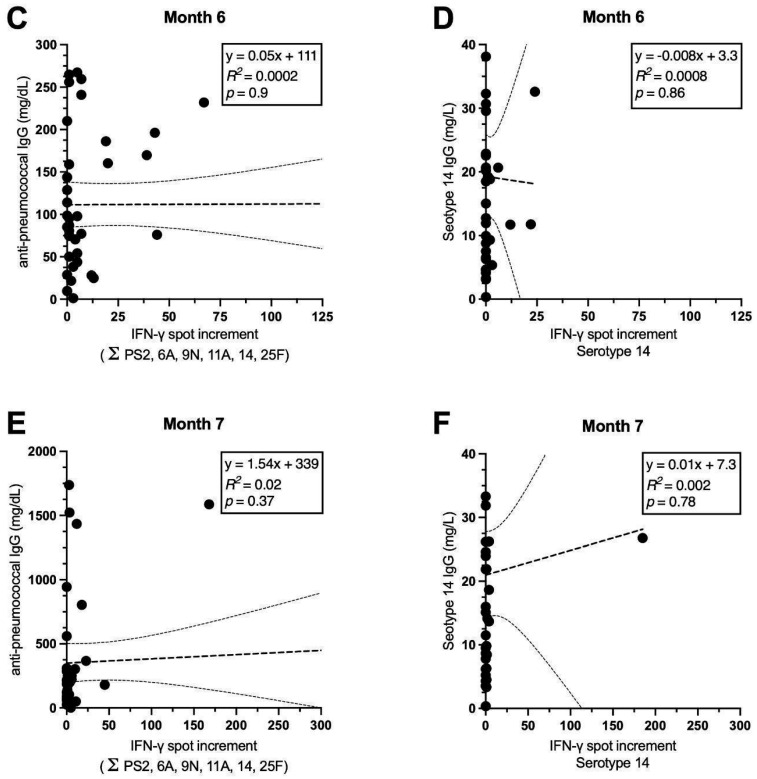
Spearman correlation analysis of the ELISpot measurements compared with IgG antibody production before and after vaccination with PCV13 and PPSV23 (*n* = 42). On the left side, IFN-γ spots are summed up for all studied serotypes on the *x*-axis. IgG antibody concentrations are displayed on the *y*-axis (**A**,**C**,**E**). On the right side, spots and IgG antibodies for PS14 are shown separately (**B**,**D**,**F**). To assess each of the ELISpot responses, median values of triplicate cultures (with antigen concentrations of 100, 150, and 200 μg/mL) are considered. The IgG antibody concentrations are measured by an IgG ELISA. The regression line is marked as a bold dashed line, while the thin dashed lines represent the 95% confidence interval.

As described in our previous paper on psoriasis patients [28], IgG antibodies increased significantly after pneumococcal vaccination. In the current study, we compared cellular and serological responses, using an IgG ELISA containing all 23 serotypes. As shown in Figure 5, IgG antibody production (against 23 serotypes) was observed in the majority of patients (A,C,E), with consistent increase throughout the surveillance period. IgG concentrations increased 7.2-fold from onset to the end of observation. Conversely, IN-γ-production did not follow the same pattern. The overall ELISpot response (sum of responses to all tested serotypes) did not change significantly. There was no correlation between the sum of ELISpot responses and IgG antibodies (*p* = 0.9 (M0), *p* = 0.9 (M6) *p* = 0.4 (M7)). The correlation coefficients remained close to zero. As previously mentioned, after vaccination, cellular responses to serotype 14 increased. As responses to serotype PS14 were rather strong, we exemplarily considered cellular and humoral responses to this serotype separately. IgG antibody production specific to serotype 14 showed a 2.5-fold increase. However, the correlation with ELISpot responses remained below statistical significance (Figure 5B,D,F). A corresponding analysis was conducted for the remaining serotypes as well but also did not show significant results.

## 4. Discussion

The objective of this study was to evaluate the cellular immunogenicity of the sequential pneumococcal vaccination protocol in psoriasis patients undergoing systemic anti-psoriatic treatment and to establish a correlation of various treatment regimens with cellular responses. In contrast to our initial expectations, we were unable to ascertain definitive evidence of an induction of a cellular immune response after vaccination in the total cohort. However, patients treated with TNF-α blockers showed significantly (*p* = 0.02) higher baseline immunity towards one of the pneumococcal serotypes, PS14, which induces an overall rather strong cellular immunity, compared to patients treated with monoclonal antibodies or methotrexate. Moreover, in patients treated with TNF-α inhibitors, we observed a median 3.5-fold increase in their specific cellular immunity towards PS14 from month 6 to 7 but not in the two other treatment groups. However, for the remaining serotypes no clear difference between the treatment groups could be established, in part due to the small sample size of patients under MTX treatment (*n* = 7). Both TNF-α-inhibitors and methotrexate were shown to downregulate CD4+-T-cells, without interfering with IL-17 and IFN-γ production in particular [29]. Interleukin as well as TNF-α inhibitors have a more targeted function regarding immunosuppression. In contrast, methotrexate acts more broadly, resulting in more complications due to impaired immune function [30,31].

Responses to the pneumococcal serotypes showed great variation, ranging from strong cellular responses for PS9N, PS14, and PS25F to an absence of reactions, particularly evident for PS6A and PS11A. Similar findings were observed in a previous study on kidney transplant recipients [32]. The results on serotype PS11A are noteworthy as they demonstrated a non-response across the entire timeline. Similar to our previous data [32], the strongest reactions were directed against the serotypes PS9N and PS14. Further research, employing a healthy control group, may offer a more comprehensive interpretation of these results.

Furthermore, the pattern of spot formation, according to the vaccine used, does not align with its respective serotype profile, which was also analyzed in detail in a previous study conducted in our facility [32]. The data of the current study indicate that there was no correlation between cellular and humoral immunity against pneumococci. With respect to humoral immune responses, however, the serotypes contained in the vaccine and the serotype-specific antibodies matched well [28]. To further analyze the discrepancy between cellular and humoral immunity, a larger cohort could be used to investigate whether strong cellular or humoral immunity is more protective against pneumococcal infection.

Assessment of successful immunization by detecting cellular reactions has been frequently and routinely used by flow cytometry or ELISpot [32]. Operating through different means of building immunity, cellular and humoral reactions are linked together particularly by the influence that T-cells have on B-cell maturation and isotype switching [27]. Despite the assumption that this interaction would lead to comparable levels of success when assessing both cellular and humoral immunity, outcomes may differ, which was also shown in previous studies. For instance, in a study of patients with psoriasis receiving methotrexate, it was observed that those vaccinated against SARS-CoV-2 exhibited a cellular reaction, while the humoral response was reduced when compared to healthy controls [33]. Similarly, other reports have noted a diminished humoral response with a preserved cellular response in immunocompromised individuals [33,34,35]. These discrepancies underscore the necessity for further investigation of cellular responses to vaccines.

In kidney transplant recipients, however, cellular and humoral reactions to sequential pneumococcal vaccination were found to be positively correlated, albeit with a relatively weak correlation coefficient [32]. The study design was comparable due to its serotype-specific measurements following the same vaccination protocol.

In a cohort of healthy individuals, Wourimaa et al. examined the cell-mediated immune response against pneumococcal antigens [36]. They demonstrated a vaccination-induced increase in IFN-γ-secreting cells in response to both protein-conjugated and polysaccharide vaccines. The study identified key differences in the stimulating pneumococcal antigens employed, which included a tetanus-toxoid and diphtheria-toxoid conjugated antigen, as well as a non-serotype-specific polysaccharide antigen. Findings indicated that individuals vaccinated with the conjugate vaccine exhibited increased IFN-γ responses to the carrier proteins (tetanus and diphtheria toxoids), suggesting activation of T-helper cells. However, after vaccination, proliferative or IFN-γ responses to pneumococcal polysaccharide antigens were non-significant. Consequently, it can be hypothesized that the response to stimulation with polysaccharide antigens, without the use of a booster, may be attributable to unspecific IFN-γ secretion, most likely by NK-cells or mechanisms related to preexisting antibodies [25,26,36]. Furthermore, recent research has underscored the importance of NK-cells for humoral and TH1 response in NK-cell-depleted mice [37].

To account for outliers, prior exposure to naturally occurring antigens has to be considered. PS14 showed considerable activity at baseline, which could be due to a higher prevalence of PS14 carriage [38,39]. We found considerable T-cell activity in response to serotype 25F, which is included in neither vaccine. This strengthens our assumption of immune-mediated cellular processes in response to pneumococcal carriage. As expected, no significant increase in cellular immunity against serotype 25F was found for kidney transplant recipients after pneumococcal vaccination [32]. Recognition of pneumococcal antigens by the innate immune system, including IFN-γ secreting cells, was shown in healthy adults. Carriage of pneumococcal antigens was linked to T-cell memory phenotypes, characterized as CD4+ CD45RO+ CCR7+/CCR7- cells, which were responsible for IFN-γ production [26]. This study highlights a dominant presence of IL-17A and TNF-α-secreting T-cells in mucosal tissue and peripheral blood and lower levels of IFN-γ with regard to pneumococcal carriage. It appears likely that IL-17A and IFN-γ play a significant role in naturally acquired protection against pneumococcal disease. Thus, there is evidence that nasopharyngeal colonization has an immunizing effect on a cellular level, potentially providing an explanation for positive cellular reaction in some of our subjects at baseline [21,26].

However, naturally acquired immunity may be in response to proteins being the target in cellular defense, rather than capsular antigens [26,40]. Together with the serotype selectivity of preexisting cellular reactions in our findings and our usage of non-protein-conjugated stimuli, the state of natural immunity against pneumococcal infection requires further investigation.

The limitations of this study must be addressed, starting with the rather small cohort. The ability to draw meaningful conclusions across treatment groups was compromised by high dropout rates and changing trends in medication protocols with methotrexate being far less frequently prescribed in favor of monoclonal interleukin inhibitors. Furthermore, patients were not excluded if vaccination against *S. pneumoniae* had previously been performed during their lifetime. In addition, our current study lacks a healthy control group. It would have been interesting to assess if discrepancies between cellular and humoral immunogenicity would have also occurred in the control group. Addressing these limitations, the impact of therapy regimens remains a subject worthy of further testing. Due to the overall low cellular responses, a definitive conclusion could not be drawn on therapy-dependent vaccination success in psoriasis patients. Previous studies highlighted a diminished effect in vaccination success in patients receiving MTX compared to TNF-α inhibitors [41], which fits well with our current data.

Both vaccines were well tolerated with no vaccine-related complications. Furthermore, there was no report of confirmed cases of pneumococcal pneumonia, leading to the assumption that the strong serological response we described in our previous paper may be sufficient for protection [28]. However, no clear statement can be made on the long-lasting effect since the majority of patients were only tested until month 1 after vaccination with PPSV23. In a subgroup of *n* = 13 patients, follow-up data at 12 months post-vaccination demonstrated a sustained and relatively stable immune response. However, due to the small sample size, a bigger cohort may be necessary for an accurate depiction.

## 5. Conclusions

In conclusion, our findings highlight the importance of measuring T-cell response when assessing the immunization process following vaccination. Although our data shows overall weak cellular reactions, humoral responses were clearly detectable. This warrants further investigation into the effects on long-term cellular immunity.

## Figures and Tables

**Table 1:** Basic patient characteristics.

Parameter ^1^	Median (Range) or Number (No. ^1^)
Median age (range), years	49 (18–67)
Patient sex (male/female)	23/19
Median interval since first diagnosis (range), years	26 (1–50)
Mean PASI (Month 0)	2.2 (0–15)
Mean DLQI (Month 0)	3.9 (0–23)
Immunosuppression, no. ^1^	
MTX > 10 mg/week	7
TNF-alpha-inhibitor (adalimumab/infliximab/etanercept/certolizumab), MTX-co-medication max. 10 mg/week)	14
IL-17-inhibitor (secukinumab/ixekizumab)	6
IL-12/23-inhibitor (ustekinumab)	4
IL-23-inhibitor (guselkumab)	11

^1^ At the time of first blood sampling. PASI, Psoriasis Area and Severity Index; DLQI, Dermatology Life Quality Index.

**Table 2:** Cellular response to sequential pneumococcal vaccination.

Serotype	2	6A	9N	11A	14	25F
Mean value of spot increments Median value of spot increments						
Month 0	2.6	0.0	11.4	0.3	8.1	11.4
*n* = 42	0.0	0.0	1.0	0.0	0.0	1.0
Month 6	4.0	0.0	8.7	0.2	1.9	7.2
*n* = 42	0.0	0.0	0.0	0.0	0.0	0.5
Month 7	3.8	0.3	12.8	0.1	5.2	6.0
*n* = 42	0.0	0.0	1.0	0.0	0.0	0.0
Month 12	4.5	1.4	5.4	1.2	3.8	4.8
*n* = 13	1.0	1.0	1.5	0.0	2.5	2.5

## Data Availability

The data presented in this study are available upon request from the corresponding author. The data are not publicly available due to privacy restrictions.

## Abbreviations

The following abbreviations are used in this manuscript:

BSA	Body Surface Area
CAP	Community-Acquired Pneumonia
CD4	Cluster of Differentiation 4
CD45RO	Cluster of Differentiation 45 Isoform RO
DLQI	Dermatology Life Quality Index
ELISA	Enzyme-Linked-Immunosorbent Assay
ELISpot	Enzyme-Linked Immunosorbent Spot Assay
IFN-γ	Interferon-γ
IgG/M	Immunoglobulin G/M
IL	Interleukin
MTX	Methotrexate
PASI	Psoriasis Area and Severity Index
PCV13	Prevenar 13
PPSV23	Pneumovax 23
PS	Pneumococcal Serotype
RKI	Robert-Koch Institute
TNF	Tumor Necrosis Factor
